# Imaging characteristics of incidentally detected cosmetic surgery-derived foreign bodies on CT images in the maxillofacial region

**DOI:** 10.1007/s11282-023-00734-2

**Published:** 2024-01-22

**Authors:** Miki Hisatomi, Yohei Takeshita, Yoshinobu Yanagi, Shunsuke Okada, Mamiko Fujikura, Suzuka Yoshida, Toshiyuki Kawazu, Junichi Asaumi

**Affiliations:** 1 Department of Oral Diagnosis and Dentomaxillofacial Radiology, Okayama University Hospital, 2-5-1 Shikata-cho, Kita-ku, Okayama, 700-8558 Japan; 2 Department of Oral and Maxillofacial Radiology, Okayama University Graduate School of Medicine, Dentistry and Pharmaceutical Sciences, 2-5-1 Shikata-cho, Kita-ku, Okayama, 700-8558 Japan; 3 Department of Dental Informatics, Okayama University Graduate School of Medicine, Dentistry and Pharmaceutical Sciences, 2-5-1 Shikata-cho, Kita-ku, Okayama, 700-8558 Japan

**Keywords:** Foreign body granuloma, Cosmetic biomaterials, Nasal prosthesis, Silicone jaw implants

## Abstract

**Objectives:**

This study examined the imaging characteristics of cosmetic surgery-derived foreign bodies in the maxillofacial region through a retrospective review of cosmetic material foreign bodies incidentally detected on computed tomography (CT) images in routine clinical practice.

**Methods:**

We retrospectively investigated cases of cosmetic surgery-derived foreign bodies other than dental materials in the maxillofacial region, using 5 years of CT image data stored on an imaging server. The imaging findings of these foreign bodies were investigated, along with patient age, patient sex, whether the foreign bodies were associated with the disease targeted by the CT scan, and the availability of cosmetic surgery information prior to examination.

**Results:**

Foreign bodies were more common in women (19/21 cases), and affected patients displayed a wide age range (20–84 years). Four types of cosmetic surgery-derived foreign bodies in the maxillofacial region were detected by CT examination: nasal prostheses (nasal region), lifting sutures and injectable facial fillers (both in the buccal region), and silicone chin implants (chin region).

**Conclusions:**

A cosmetic surgery-derived foreign body should be suspected when a foreign body is identified without a dental source of infection. In addition, cosmetic surgery-derived foreign bodies may be present in numerous patients, regardless of age or sex.

## Introduction

Various materials are used in cosmetic surgery, and it is challenging to maintain extensive knowledge about them. In dentistry, surgical orthodontic treatments (e.g., osteotomy and otoplasty) for jaw deformities and dysarthria often produce significant esthetic improvements, as well as improvements in oral function [1, 2]. These treatments are regarded as dental care; thus, they are very familiar to dentists. However, cosmetic surgery performed as medical treatment is less familiar to dentists, and they may have difficulty diagnosing the underlying problem. Furthermore, patients tend to withhold details of such procedures; they are unlikely to self-report a history of cosmetic surgery during an initial dental examination. Occasionally, cosmetic materials become a source of infection; subjective symptoms (e.g., swelling and pain at the surgical site) may be observed, and imaging studies may reveal that cosmetic surgery was performed in the same area. Although there have been sporadic reports of these foreign body granulomas [3–6], dentists generally have minimal experience with cosmetic surgery-derived foreign bodies; these foreign bodies are not fully understood, despite their potential impact on detection of the main lesion.

Therefore, clinicians engaged in imaging interpretation should understand the types of cosmetic surgery-derived foreign bodies that they may encounter when analyzing images of the maxillofacial region, as well as the imaging characteristics of such foreign bodies. This study examined the imaging characteristics of cosmetic surgery-derived foreign bodies in the maxillofacial region through a retrospective review of cosmetic material foreign bodies incidentally detected on computed tomography (CT) images in routine clinical practice. This presentation of the imaging findings of multiple cosmetic materials may prevent misdiagnosis during interpretation of maxillofacial images.

## Materials and methods

This study included cases of head-and-neck diseases that were examined by CT at Okayama University Hospital Dentistry during the 5-year period from 2017 to 2021. Using CT image data stored on the image server, we retrospectively investigated cases involving cosmetic surgery-derived foreign bodies other than dental materials in the maxillofacial region. For each cosmetic surgery-derived foreign body included within the imaging area, the location, shape, CT values, and imaging findings of surrounding tissue were obtained from CT images.

Images were acquired using four different types of CT scanner: Aquilion ONE (Canon Medical Systems Corporation, Tochigi, Japan); Aquilion Precision (Canon Medical Systems Corporation, Tochigi, Japan); Discovery CT750 HD (GE Healthcare, Milwaukee, WI, USA); and SOMATOM Definition Flash (Siemens, Nurnberg, Germany). CT scans were obtained with the following parameters: field of view: 12.9 × 12.9 to 32 × 32 cm; tube voltage: 120–140 kV; tube current: 150–500 mA.

All CT images taken multiple times in the same patient during the study period were also examined. The total number of CT cases over the 5-year period from 2017 to 2021 was 5951. The foreign bodies other than normal anatomy that were observed as high-density areas within the imaging range of the maxillofacial region were targeted using CT soft tissue images. CT images of the full extent of the head and neck were reviewed by one dental radiologist. When the same site was treated multiple times and the amount of foreign body changed, only the first image was included; this was regarded as one case. Foreign bodies at different sites in the same patient were regarded as separate cases. Cases were excluded if they met any of the following criteria: foreign bodies of dental material origin (e.g., plates and screws associated with jaw deformities), foreign bodies introduced via trauma, vascular calcifications, and calcifications on the skin.

CT values were measured using a high-resolution medical image monitor (EIZO Corporation, Ishikawa, Japan). The region of interest area for CT value measurements was established in a freehand manner at the center of the lesion, approximately 1–2 mm from the lesion margin. The mean CT value between initial value and the remeasured value at 2 weeks was defined as the CT value of one observer. CT value assessments were performed by two observers with experience in CT examinations and CT measurement (10 and 26 years of experience, respectively).

We also obtained information regarding any additional useful imaging findings by other modalities such as panoramic radiography, magnetic resonance imaging (MRI), and ultrasonography during the same period. Finally, we investigated age, sex, whether the foreign bodies were associated with the disease targeted by the CT scan, and whether any information about cosmetic surgery was available prior to examination.

This study was performed in accordance with the principles of the Declaration of Helsinki. Approval was granted by the Ethics Committee of Okayama University (Date: 5 August 2022/No. 2208-038).

## Results

**Fig. 1:**
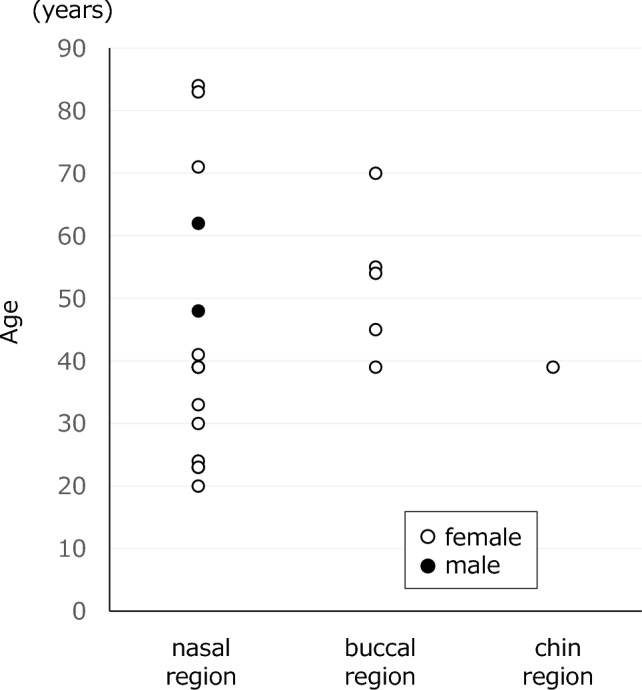
Patient ages and lesion locations: Cosmetic surgery-derived foreign bodies in the maxillofacial region were more common in women (90.5%, 19/21 cases), and the age range varied from 20 to 84 years. The two foreign bodies observed in the male were both in the nasal region

In total, there were 21 cases involving four types of cosmetic surgery-derived foreign bodies in the maxillofacial region identified via CT examination. There were 15 cases (one type) in the nasal region, five cases (two types) in the buccal region, and one case (one type) in the chin region. Their ages and sexes are shown in Fig. 1.

**Fig. 2:**
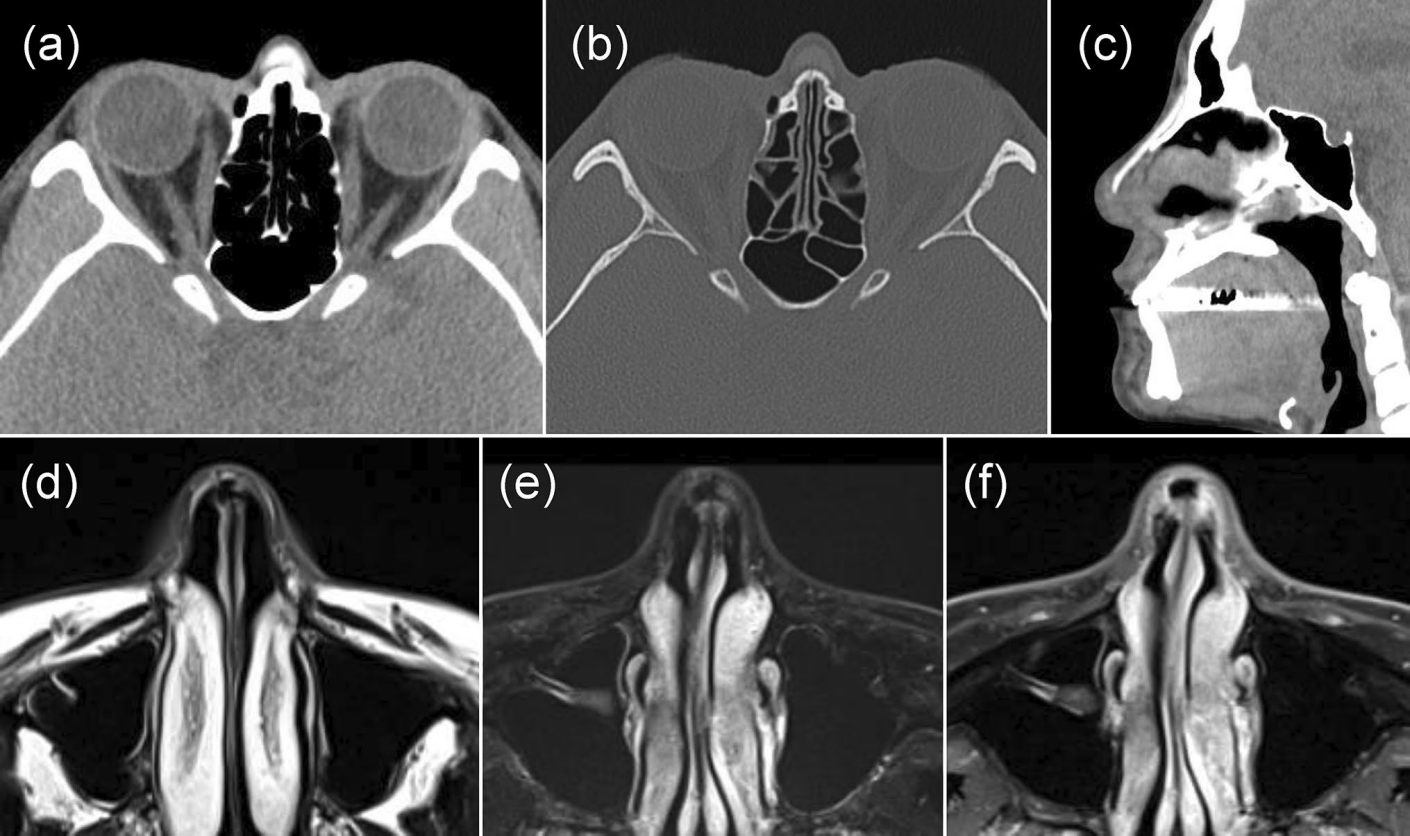
Foreign body in the nasal region. A foreign body comprising a suspected nasal prosthesis was observed on the nasal dorsum. The foreign material showed an I-shaped high-density area with a CT value of 218 HU (**a**: axial soft tissue condition, **b**: axial bone condition, **c**: sagittal soft tissue condition). It showed no signal intensity on T1-weighted (**d**) and STIR (**e**) images, and no contrast enhancement on contrast-enhanced T1-weighted images (**f**)

**Fig. 3:**
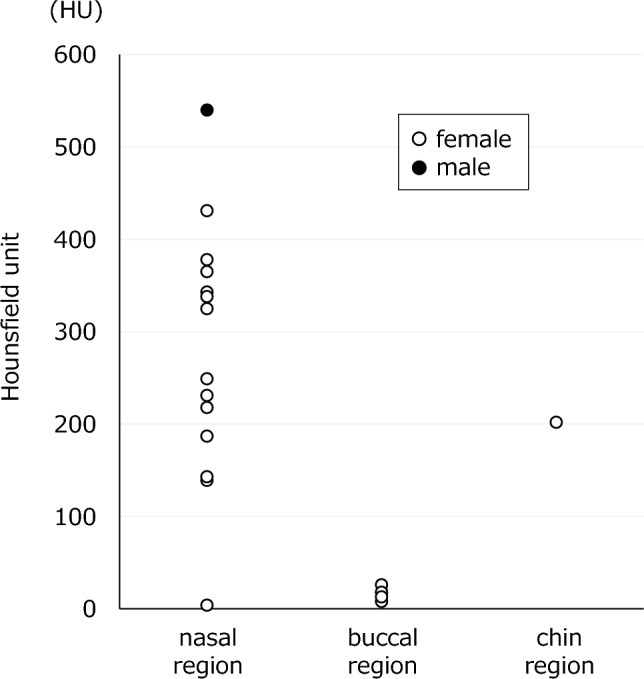
Computed tomography values (in Hounsfield units) of lesions: Cosmetic surgery-derived foreign bodies in the nasal region were observed over a wide range of 4–540 HU, suggesting that various materials were used. On the contrary, the buccal foreign bodies showed a value of 8–26 HU, which is similar to that of water, and an injection agent made of a material similar to water was suspected

All the 15 cases in the nasal region were located on the nasal dorsum. In terms of shape, 13 cases had a straight I-shape and two cases had an L-shape; thus, the 15 cases had a rod-like morphology Fig. 2. The mean CT value of the 14 cases was 278.0 Hounsfield units (HU; range: 4–540 HU) Fig. 3. One male case was plate like and CT values could not be determined, but the density was uniform on soft tissue images. Hard tissue images showed homogeneous density in 12 cases, but three cases displayed homogeneous density surrounded by an osteosclerotic margin. Regarding other modalities, panoramic radiographs showed no obvious findings, although increased opacity in the median nasal area was suspected in some cases. Six cases had relevant findings on MRI. In five cases, nasal foreign bodies showed signal voids on T1-weighted and STIR images, similar to air-containing areas; there was no contrast enhancement. The remaining case demonstrated moderate signal intensity on T1-weighted images (similar to the intensity of muscle) and a uniform watery high signal on T2-weighted images (similar to the signal of cerebrospinal fluid). This case had a CT value of 4 HU. For all the 15 cases in the nasal area, no cosmetic surgery was declared prior to examination; the foreign body was found incidentally, unrelated to the purpose of CT examination.

**Fig. 4:**
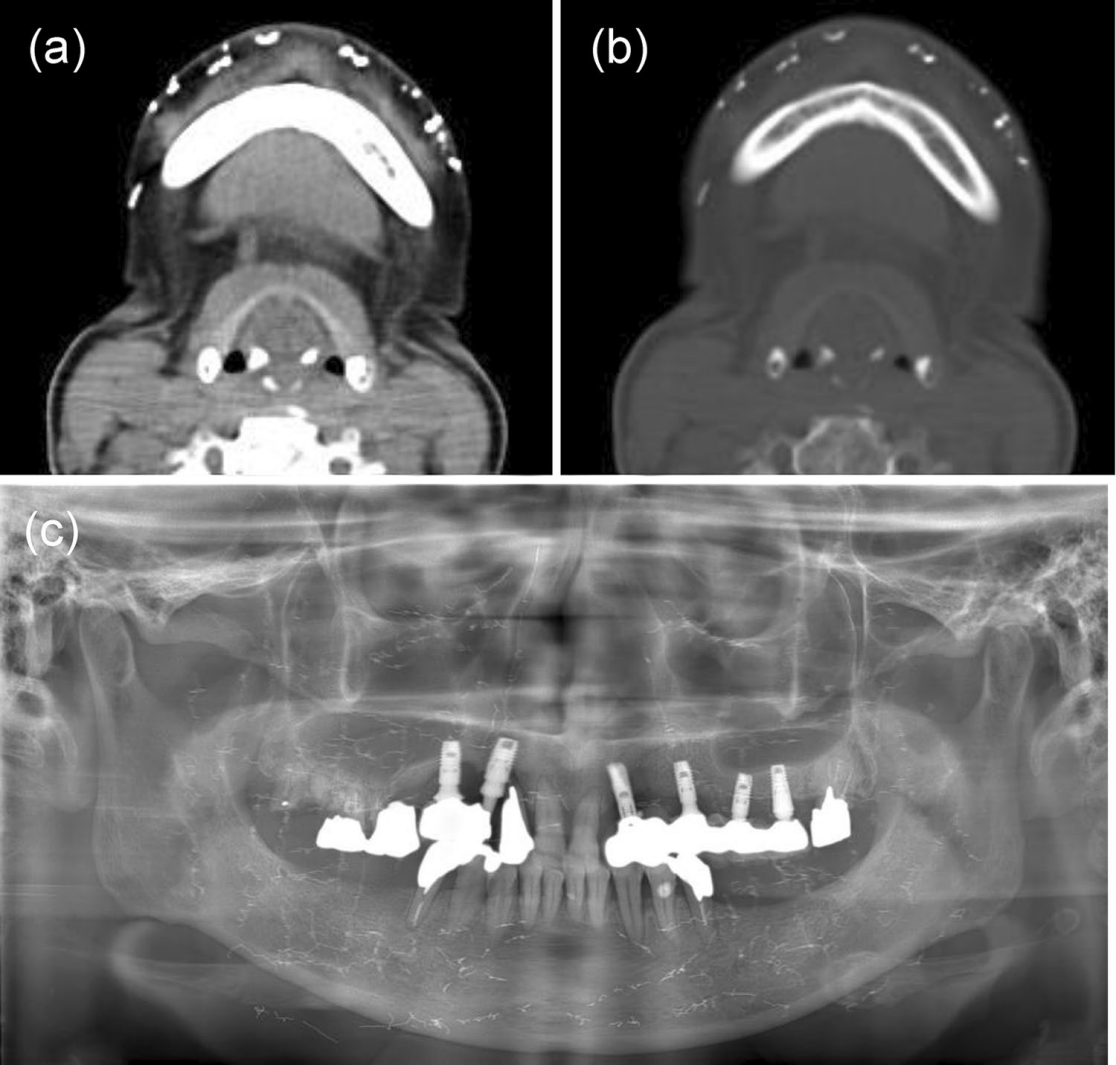
Foreign body in the buccal region. Multiple metallic thread-like linear foreign bodies were detected under the buccal skin (**a**: axial soft tissue condition, **b**: axial bone condition). Panoramic radiographs showed numerous thread-like opacities overlapping from the maxillary sinus to the maxilla and throughout the mandible, but not in the orbit or pyriform aperture (**c**)

**Fig. 5:**
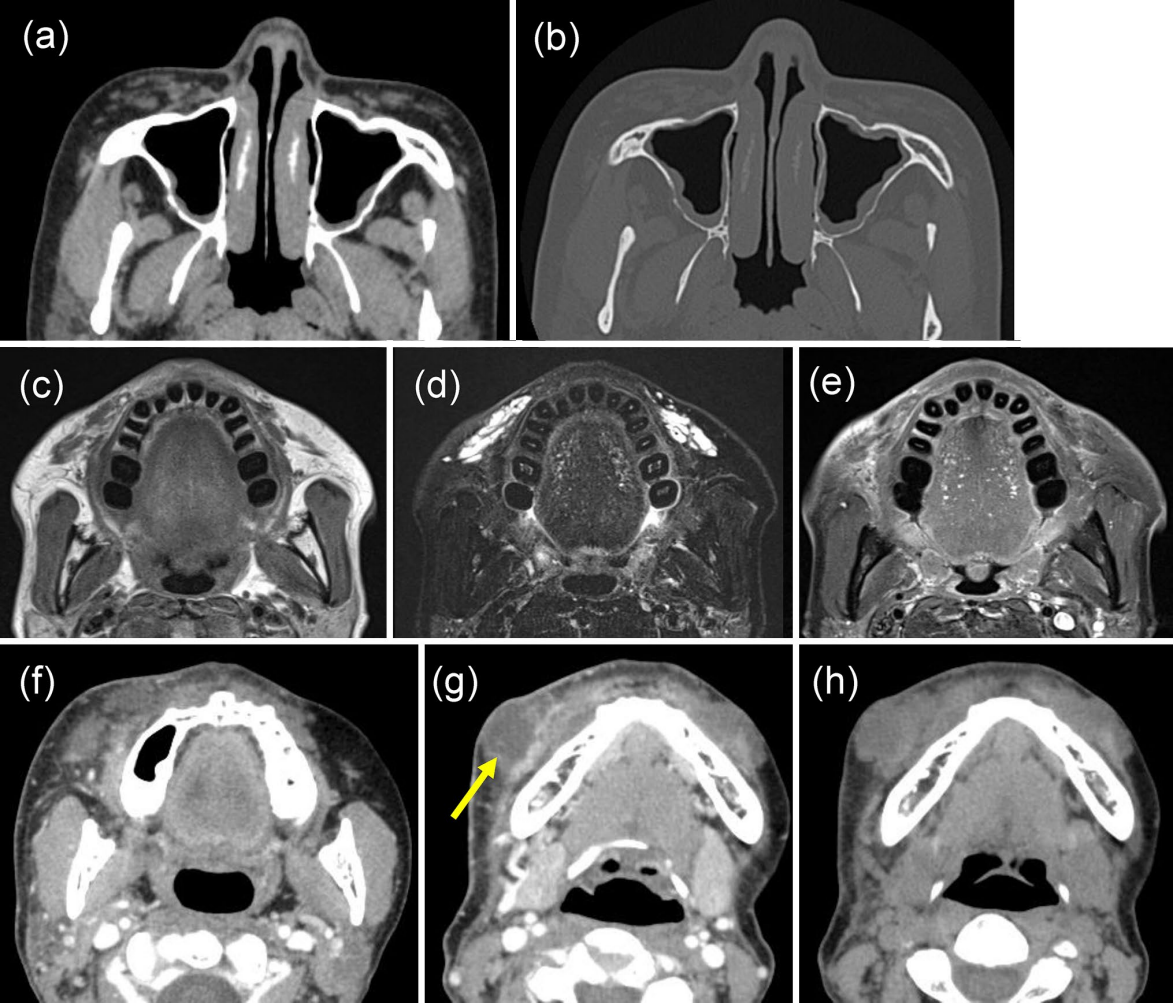
Foreign body in the buccal region. Injectable fillers comprising multiple irregularly shaped, cord-like injections were present in the bilateral buccal region. CT soft tissue images (**a**) were readable, but the bone condition was poorly delineated (**b**). T1-weighted images (**c**) showed moderate signal intensity and STIR images (**d**) showed high signal intensity; contrast T1-weighted images (**e**) showed no contrast enhancement. A 54-year-old woman with a history of Aquamid^®^ (**f**, **g**); right-sided buccal swelling was evident, with a large mass (**f**). In another image, an abscess (arrow) without internal contrast was observed (**g**). A foreign body was also observed in the fat layer within the left buccal region, and the presence of a high-density cosmetic surgery-derived foreign body was suspected (**h**)

In the buccal region, five foreign bodies (two types) were detected. In one case, multiple metallic thread-like linear foreign bodies were present under the buccal skin (Fig. 4). These foreign bodies were linear and CT values could not be obtained. Concerning other modalities, panoramic radiographs showed numerous thread-like opacities overlapping from the maxillary sinus to the maxilla and throughout the mandible, but not in the orbit or pyriform aperture. In four cases, the lesions comprised irregularly shaped lumps or cords extending from the zygomaticus muscle to the subcutaneous fat layer; they were quite visible (Fig. 5). The mean CT value of these four buccal foreign bodies was 16.3 HU (range: 8–26 HU) (Fig. 3). With respect to other modalities, panoramic radiographs showed no obvious findings. MRI showed moderate signal intensity on T1-weighted images and high signal on STIR images. No contrast enhancement was noted. In all five cases of buccal foreign bodies, no cosmetic surgery was declared prior to examination. In both of the two cases where CT findings suggested a cosmetic surgery-derived foreign body as the infectious source of the inflammatory findings, an additional patient interview confirmed a history of Aquamid^®^ injection approximately 2 years earlier (Fig. 5). In the remaining three cases, the foreign body was discovered incidentally, unrelated to the purpose of the CT scan.

**Fig. 6:**
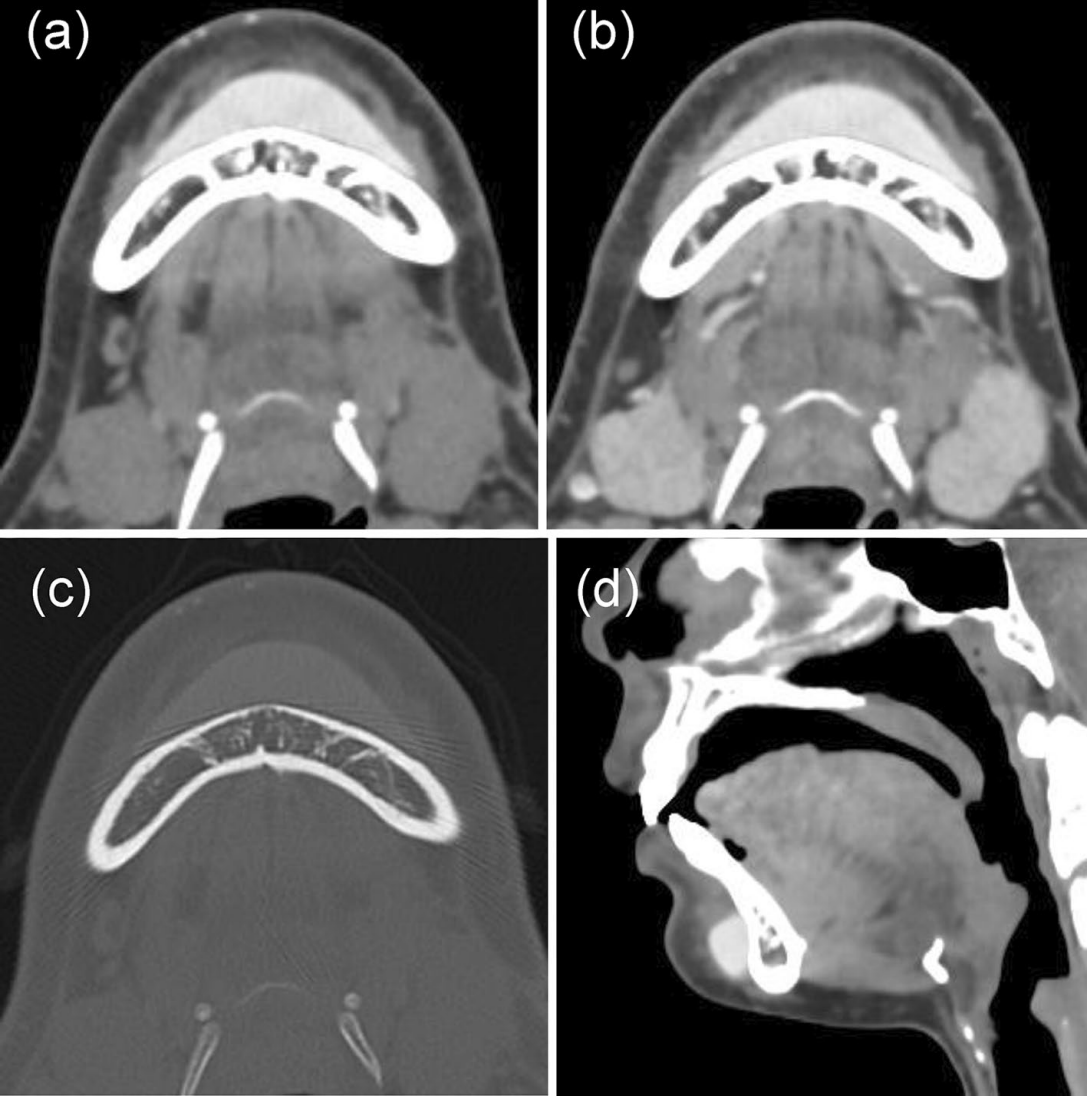
Foreign body in the chin region. A foreign body (presumably a silicone jaw implant) was identified in the chin region. On CT soft tissue images, the foreign body showed a uniform high-density area of 202 HU (**a**) and no contrast enhancement (**b**). In terms of bone condition, the morphology of the foreign body was visible, but its density was low and delineation was poor (**c**). Sagittal images showed a clear, well-defined jaw formation (**d**)

The foreign body in the chin region showed a hemispherical morphology with smooth edges. The foreign body exhibited a uniform high-density area of 202 HU without contrast enhancement (Fig. 6). Regarding other modalities, panoramic radiographs showed no abnormal findings in the chin region. In the case involving the chin region, no cosmetic surgery was declared prior to examination; the detection of foreign bodies was incidental and unrelated to the disease targeted by the CT scan.

## Discussion

Cosmetic surgery for esthetic purposes is an unrestricted medical procedure with diverse treatment methods. Therefore, diverse cosmetic biomaterials are used in such procedures. In this study, we identified 21 cases involving four types of cosmetic surgery-derived foreign bodies in the nasal, buccal, and chin regions of the face that were included in CT scans.

### Foreign body in the nasal region

Cosmetic rhinoplasty can change the height and shape of the nose. When the nose height is altered, the height of the nasal tip or bridge of the nose can be modified. With respect to reshaping, the shape of the nasal tip or width of the nose can be reduced; the nostril shape can be changed. Foreign bodies in the nasal area from these procedures can include prostheses, injectables, and sutures composed of polydioxanone. Dorsal rhinoplasty using alloplastic implant materials, such as silicone, is the rhinoplasty procedure most commonly performed in Asian populations [7]. The artificial cartilage is constructed from silicone resin, and it exhibits either I- or L-shape, consistent with the imaging findings in the present study.

Of the 15 nasal foreign bodies detected during the study period, 14 had CT values greater than 100 HU and were regarded as nasal prostheses. Soft tissue images showed a high-density, bone-like material that was easily detected. The remaining foreign body, which was difficult to detect on soft tissue images, had a CT value of 4 HU and high signal intensity on STIR images; it presumably comprised a water-like injectable material that had been injected in a linear manner into the nasal dorsum. On hard tissue images, it was difficult to detect all foreign bodies in the nasal dorsum, but there were three foreign bodies with a heterogeneous and high-density posterior margin. Although most nasal foreign bodies exhibited uniform density, various alloplastic implant materials are available [8]; thus, compositional differences among products were suspected. Silicone plates were clearly visible as very low-intensity plates (void signals) on sagittal or coronal T1- and T2-weighted MRI [9]. In five of the six cases where a foreign body was present in the nasal region and included in the scope of MRI, the foreign body displayed a void signal suggesting that the material was silicone. In the present study, there were no complications after cosmetic rhinoplasty (e.g., infection), which may partially explain the lack of self-reporting.

### Foreign body in the buccal region

Two different types of foreign bodies (five cases in total) were found in the buccal region. Thread lifting with sutures is a cosmetic procedure where lax tissue is lifted and repositioned to create a more youthful-looking facial contour [10]. Lifting uses absorbable threads such as polydioxanone, polycaprolactone, and poly L-lactic acid threads, as well as nonabsorbable threads comprising Prolene^®^ and polyproline [11]. In one case in the present study, a nonabsorbable thread was suspected, but the material could not be identified. On CT images, it was difficult to determine the continuity of the foreign body, which appeared to be multiple punctate foreign bodies. However, panoramic radiographs were useful in identifying the history of thread lifting because the foreign bodies were linear and clearly visible across a large portion of the buccal region, excluding the nasal and orbital areas.

In the buccal region, cosmetic surgery is performed to lift and improve skin from within through the application of injectables into wrinkles and skin depressions. Commonly injected sites on the face include the perioral area, periocular region, nasolabial folds, malar fat pad, marionette lines, glabella, and lips [12]. There are numerous types of injectable facial fillers, including autologous fat fillers, collagen fillers, calcium hydroxyapatite fillers, hyaluronic acid fillers, poly-l-lactic acid, polyalkylimide and polyacrylamide hydrogels, and silicone oil fillers [12, 13]. Although hyaluronic acid is the most widely used and well-known injectable agent, its effect lasts only 1 to 2 years, and it requires regular visits. Thus, some cosmetic surgeons use the semi-permanent Aquamid^®^ for economic reasons. Aquamid^®^ is a transparent, nonabsorbable injectant consisting of 97.5% pure water and 2.5% cross-linked polyacrylamide [14]. However, nonabsorbable preparations are foreign bodies that have higher risks of secondary infection.

In the present study, postoperative infections occurred in two of the four cases involving buccal injections. The two patients who desired treatment of buccal swelling did not self-report a history of cosmetic surgery during the initial examination. Because imaging findings suggested that cosmetic materials were the source of infection in these two patients, they were reinterviewed after imaging; both had a history of semi-permanent residual Aquamid^®^ injection approximately 2 years prior (Fig. 5). In clinical settings, reactive lesions can easily be misdiagnosed as soft tissue tumors or cysts. Because few problems are reported after cosmetic procedures and there is limited experience managing such problems, especially in rural areas, appropriate diagnoses can be difficult to establish. When a foreign body is detected without a dental source of infection, a history of cosmetic surgery should be considered; the foreign body may have a cosmetic origin.

CT images of foreign bodies in the buccal region showed an irregularly shaped lumpy or cord-like shape from the zygomatic muscle to the subcutaneous fat layer, with multiple foreign bodies. CT images of the foreign bodies revealed soft tissue-like density similar to muscle, which could be overlooked without careful interpretation. When inflammatory findings were observed without a dental source of infection, a cosmetic surgery-derived foreign body was the possible cause.

MRI showed high signal intensity on T2-weighted and STIR images, indicating that water was the main component of the foreign body, which aided in diagnosis. Therefore, additional MRI examination may be useful in the cases of unexplained inflammation that are difficult to diagnose because they can be identified by T2-weighted or STIR images.

### Foreign body in the chin region

A prominent, well-defined chin and jawline are key aspects of a harmonious, beautiful face. Contouring of the lower third of the face is an important component of cosmetic surgery [15]. Chin augmentation can be performed with injectables, implantation, or osseous genioplasty [15]. Silicone jaw implants are frequently used in cosmetic surgery to emphasize facial harmony [16, 17]. The foreign body identified in the chin region in the present study exhibited uniform density and was detected incidentally. The silicone jaw implants in the chin region had a smooth hemispherical shape; after initial recognition, diagnosis was relatively easy for oral radiologists.

Cosmetic surgery-derived foreign bodies in the maxillofacial region were more common in women (90.5%, 19/21 cases), and the age range varied from 20 to 84 years. In two cases where the foreign body was regarded as a possible source of infection, reinterviewing revealed a history of cosmetic surgery; in all cases, regardless of sex or site, there was no self-reporting during the initial examination. Therefore, clinicians should be aware that cosmetic surgery-derived foreign bodies may be present in numerous patients, regardless of age or sex.

This study had some limitations. First, because this study focused on cosmetic surgery-derived foreign bodies in the maxillofacial region that were incidentally detected on CT images, injectable agents in soft tissues (e.g., the lips) may have been missed. Although MRI may be useful to identify injectables because of their high signals on STIR and T2-weighted images, it was not realistic in the present study because the number of MRI examinations was much smaller than the number of CT examinations. Second, unlike urban areas where cosmetic surgery is commonly performed, the number of foreign bodies associated with cosmetic surgery was estimated to be low in the rural area where this study was conducted; this regional difference may have influenced the findings. Third, it was difficult to identify all foreign bodies, which made diagnosis challenging because individuals who underwent cosmetic surgery may have forgotten or denied the procedure; alternatively, they may not have known the type of foreign material used.

In conclusion, we observed the following four types of cosmetic surgery-derived foreign bodies in the maxillofacial region that could be identified via CT examination: nasal prostheses (nasal region), lifting sutures and injectable facial fillers (both in the buccal region), and silicone chin implants (chin region). Foreign bodies in the buccal region included a case in which the foreign body was the source of infection; a cosmetic surgery-derived foreign body should be suspected when a foreign body is identified without a dental source of infection. In addition, clinicians should be aware that cosmetic surgery-derived foreign bodies may be present in numerous patients, regardless of age or sex.
